# Connect MDS/AML: design of the myelodysplastic syndromes and acute myeloid leukemia disease registry, a prospective observational cohort study

**DOI:** 10.1186/s12885-016-2710-6

**Published:** 2016-08-19

**Authors:** David P. Steensma, Medrdad Abedi, Rafael Bejar, Christopher R. Cogle, Kathryn Foucar, Guillermo Garcia-Manero, Tracy I. George, David Grinblatt, Rami Komrokji, Xiaomei Ma, Jaroslaw Maciejewski, Daniel A. Pollyea, Michael R. Savona, Bart Scott, Mikkael A. Sekeres, Michael A. Thompson, Arlene S. Swern, Melissa Nifenecker, Mary M. Sugrue, Harry Erba

**Affiliations:** 1 Adult Leukemia Program, Dana-Farber Cancer Institute, Boston, MA USA; 2 Division of Hematology and Oncology, University of California, Davis, Comprehensive Cancer Center, Sacramento, CA USA; 3 Division of Hematology and Oncology, University of California, San Diego, Moores Cancer Center, La Jolla, CA USA; 4 Division of Hematology and Oncology, Department of Medicine, University of Florida, Gainesville, FL USA; 5 Department of Pathology, University of New Mexico, Albuquerque, NM USA; 6 Department of Leukemia, Division of Cancer Medicine, The University of Texas MD Anderson Cancer Center, Houston, TX USA; 7 Hematology, North Shore University Health System, Evanston, IL USA; 8 Medical Oncology, Moffitt Cancer Center, Tampa, FL USA; 9 Yale School of Public Health, New Haven, CT USA; 10 Department of Translational Hematology and Oncology Research, Cleveland Clinic Foundation, Cleveland, OH USA; 11 Division of Hematology, University of Colorado Cancer Center, Aurora, CO USA; 12 Division of Hematology/Oncology, Vanderbilt University Medical Center/Vanderbilt-Ingram Cancer Center, Nashville, TN USA; 13 Clinical Research Division, Fred Hutchinson Cancer Research Center, Seattle, WA USA; 14 Department of Hematology and Oncology, Cleveland Clinic Foundation, Cleveland, OH USA; 15 Aurora Research Institute, Aurora Health Care, Milwaukee, WI USA; 16 Celgene Corporation, Summit, NJ USA; 17 Division of Hematology and Oncology, University of Alabama at Birmingham, Birmingham, AL USA

**Keywords:** Myelodysplastic syndromes, Acute myeloid leukemia, Idiopathic cytopenia of undetermined significance, Registry, Treatment patterns, Clinical outcomes, Patient-reported outcomes, Biomarkers, Clonal hematopoiesis of indeterminate potential (CHIP)

## Abstract

**Background:**

Myelodysplastic syndromes (MDS) and acute myeloid leukemia (AML) are myeloid neoplasms in which outgrowth of neoplastic clones disrupts normal hematopoiesis. Some patients with unexplained persistent cytopenias may not meet minimal diagnostic criteria for MDS but an alternate diagnosis is not apparent; the term idiopathic cytopenia of undetermined significance (ICUS) has been used to describe this state. MDS and AML occur primarily in older patients who are often treated outside the clinical trial setting. Consequently, our understanding of the patterns of diagnostic evaluation, management, and outcomes of these patients is limited. Furthermore, there are few natural history studies of ICUS. To better understand how patients who have MDS, ICUS, or AML are managed in the routine clinical setting, the Connect MDS/AML Disease Registry, a multicenter, prospective, observational cohort study of patients newly diagnosed with these conditions has been initiated.

**Methods/Design:**

The Connect MDS/AML Disease Registry will capture diagnosis, risk assessment, treatment, and outcomes data for approximately 1500 newly diagnosed patients from approximately 150 community and academic sites in the United States in 4 cohorts: (1) lower-risk MDS (International Prognostic Scoring System [IPSS] low and intermediate-1 risk), with and without del(5q); (2) higher-risk MDS (IPSS intermediate-2 and high risk); (3) ICUS; and (4) AML in patients aged ≥ 55 years (excluding acute promyelocytic leukemia). Diagnosis will be confirmed by central review. Baseline patient characteristics, diagnostic patterns, treatment patterns, clinical outcomes, health economics outcomes, and patient-reported health-related quality of life will be entered into an electronic data capture system at enrollment and quarterly for 8 years. A tissue substudy to explore the relationship between karyotypes, molecular markers, and clinical outcomes will be conducted, and is optional for patients.

**Discussion:**

The Connect MDS/AML Disease Registry will be the first prospective, observational, non-interventional study in the United States to collect clinical information, patient-reported outcomes, and tissue samples from patients with MDS, ICUS, or AML receiving multiple therapies. Results from this registry may provide new insights into the relationship between diagnostic practices, treatment regimens, and outcomes in patients with these diseases and identify areas for future investigation.

**Trial registration:**

Connect MDS/AML Disease Registry (NCT01688011). Registered 14 September 2012.

**Electronic supplementary material:**

The online version of this article (doi:10.1186/s12885-016-2710-6) contains supplementary material, which is available to authorized users.

## Background

Myelodysplastic syndromes (MDS) are a heterogeneous group of clonal myeloid malignancies characterized by ineffective hematopoiesis, peripheral blood cytopenias, and a propensity to transform into acute myeloid leukemia (AML) [1–3]. AML, which can arise de novo or secondary to prior myeloproliferative neoplasms or MDS, is defined by ≥ 20 % myeloid blasts in the marrow or blood, or the presence of specific cytogenetic abnormalities [4]. In the elderly, AML and MDS tend to have similar presentation with cytopenias and associated clinical manifestations of these cytopenias, including infection, bleeding, and the poor oxygen-carrying capacity characteristic of anemia [2, 5]. MDS and AML are classified using World Health Organization criteria based on blood counts, morphological criteria, and cytogenetic data [6, 7]. Some patients with persistent cytopenia(s) may not meet the minimal diagnostic criteria for MDS, yet no other diagnosis is apparent [8]. These patients are said to have idiopathic cytopenia of undetermined significance (ICUS) [8]. Unlike monoclonal gammopathy of undetermined significance, a clonal state that is a precursor to multiple myeloma or other plasma cell neoplasms, ICUS is not a clonal disorder by definition; if a clonal mutation in a myeloid neoplasia–associated gene is present, the patient is instead said to have clonal hematopoiesis of indeterminate potential (CHIP) [9]. Although some patients with ICUS may eventually develop MDS or AML, the proportion that do is unknown as there is a lack of natural history studies of this condition [9–11].

Although MDS, ICUS, and AML can occur at any age, they are most common in older patients. In the United States, the median age at diagnosis of MDS and AML is approximately 70 years, although exact estimates vary [12, 13]. Less is known regarding the epidemiology of ICUS, although the median age reported in the few existing studies ranges from 61 to 69 years [11].

Because of varying criteria for diagnosis, omission of MDS in cancer registries until recently, and incomplete evaluation of many elderly patients with mild cytopenias, it has been difficult to accurately assess the incidence and prevalence of MDS [2, 14, 15]. Based on US Medicare claims data, it is estimated there may be ≥ 75 new cases of MDS per 100,000 people aged ≥ 65 annually in the United States, making MDS one of the most common hematologic malignancies [2, 15, 16]. AML is the most common acute leukemia in the United States, with approximately 20,000 new cases annually [12]. The incidence of ICUS remains unclear.

MDS treatment recommendations are based on individual patient characteristics and disease risk, which can be assessed using one of several prognostic scoring systems [17], such as the widely used 1997 International Prognostic Scoring System (IPSS). Patients with asymptomatic lower-risk (IPSS low/intermediate-1 [Int-1] risk) MDS are often monitored using a “watch and wait” approach, without specific therapy [18]. Patients with symptomatic lower-risk MDS are generally treated with low-intensity therapies such as supportive care (ie, transfusion support or erythropoiesis-stimulating agents) or lenalidomide, whereas those with higher-risk (IPSS int-2/high risk) MDS are often treated more intensively with disease-modifying therapy, including the DNA hypomethylating agents (HMAs) azacitidine or decitabine, cytotoxic chemotherapy, or allogeneic stem cell transplant [17]. However, except in younger, healthier patients who can potentially be cured with allogeneic stem cell transplant, MDS therapy is largely palliative, and many questions remain regarding the optimal management of patients with MDS [2].

The IPSS was revised (IPSS-R) in 2012 to improve risk stratification [19]. The IPSS-R includes more parameters than the IPSS and adds a fifth intermediate risk category that doesn’t fall cleanly into lower or higher risk, which may make this fifth category challenging for physicians to incorporate into routine clinical practice [2, 17, 19]. Additionally, a modification of the IPSS-R that incorporates mutational data was recently proposed, providing enhanced predictive power in patients with MDS across the course of the disease, regardless of treatment history [20]. Although the IPSS-R was designed to improve prognostic classification, prospective studies detailing how the IPSS-R is being used in clinical practice and what effect this has on real-world treatment decisions and outcomes have yet to be conducted.

AML treatment recommendations are largely based on age, with intensive chemotherapy and transplant generally reserved for patients less than 70 years of age [5]. Patient fitness is also taken into account when determining eligibility for intensive treatment and transplant [21], and this often requires clinicians to make difficult judgments. Treatment options for older patients with AML who are not eligible for intensive treatment are limited and, outside of a clinical trial, typically include HMAs, low-dose cytarabine, and supportive care [5, 12]. Outcomes in older patients with AML remain dismal, with a 5-year survival rate of 5 % in patients > 65 years of age in the United States, which lags behind the 38 % 5-year survival rate in patients < 65 years of age [5, 12].

Although various guidelines exist for the treatment of patients with MDS and AML, such as those of the National Comprehensive Cancer Network (NCCN) or the European LeukemiaNet [17, 21–23], patterns of treatment and clinical outcomes in patients with MDS or AML outside of clinical trials are poorly characterized. Treatment decision-making can be complex and challenging, especially for elderly patients who may have comorbid conditions and poor performance status [5, 24]. Moreover, a recent survey of physicians treating patients with MDS indicated that they frequently did not adhere to NCCN guidelines for length of treatment [25].

There are currently no specific treatments for ICUS other than addressing factors contributing to cytopenias when identified, and it is recommended that patients be monitored with regular follow-up hematologic assessments to surveil for progression to an overt myeloid neoplasm [11]. No large observational studies have been performed to date to inform best clinical practices and to understand long-term outcomes in patients with ICUS [9, 11].

There are currently gaps in the knowledge of MDS, ICUS, and AML with regard to diagnostic trends, prognostic categorization, long-term treatment patterns, and clinical and health-related quality of life (HRQOL) outcomes. Moreover, data from patients enrolled in clinical trials may not apply to patients treated outside of a trial, such as those who may lack sufficient resources to travel to participate in a trial or who are excluded from such trials due to poor performance status and multiple comorbidities [5, 26, 27]. The Connect MDS/AML Disease Registry (Clinicaltrials.gov Identifier NCT01688011) is a multicenter, prospective, observational cohort study of patients with newly diagnosed MDS, ICUS, or AML in the United States. This registry aims to acquire robust data that will be representative of these patient populations in the United States. It is designed to capture patterns of diagnosis, risk assessment, and treatment of MDS, ICUS, and AML as well as clinical and patient-reported outcomes (PROs). This registry represents an opportunity to document key variables affecting treatment decisions and clinical outcomes in MDS, ICUS, and AML and to provide new insight into these heterogeneous diseases. The primary objectives of the disease registry are to:

1. Describe the current and evolving patterns of diagnosis, prognosis, evaluation, treatment, clinical monitoring, and outcome measures;
2. Compare actual clinical practice patterns in both community and academic settings with existing management guidelines (eg, NCCN);
3. Describe treatment patterns and the associated short- and long-term outcomes in non-del(5q) patients and in del(5q) patients with or without additional cytogenetic abnormalities, including response, safety, disease progression, and survival;
4. Summarize PROs (eg, HRQOL) and health economics and outcomes research (HEOR) and their association with patient characteristics, treatment regimens, and clinical outcomes.

The disease registry study plan also includes a correlative substudy designed to identify molecular markers and evaluate their potential impact on prognostication and/or treatment outcomes.

## Methods/Design

The Connect MDS/AML Disease Registry was designed collaboratively by 2 scientific steering committees (SSCs) composed of academic and community-based practitioners in MDS (SSC-MDS) and AML (SSC-AML) in partnership with Celgene Corporation. The SSCs include experts in molecular and correlative research, as well as HRQOL, and are responsible for managing the study with guidance and review by Celgene Corporation.

### Setting

Patients will be enrolled at approximately 150 sites in the United States. Hematologists or oncologists experienced in the treatment of MDS, ICUS, or AML expressing an interest in participating in the disease registry will be evaluated as site principal investigators. Study investigators must maintain a practice with enough potential patients to achieve the quarterly enrollment target, and have adequate staff available for coordinating the study and conducting daily research activities. To best capture the distribution of routine clinical practice settings in which patients are typically treated, approximately 70 to 80 % of the sites will be community-based clinics and 20 to 30 % will be academic institutions (defined as affiliated with a medical school).

### Sample size

**Fig. 1:**
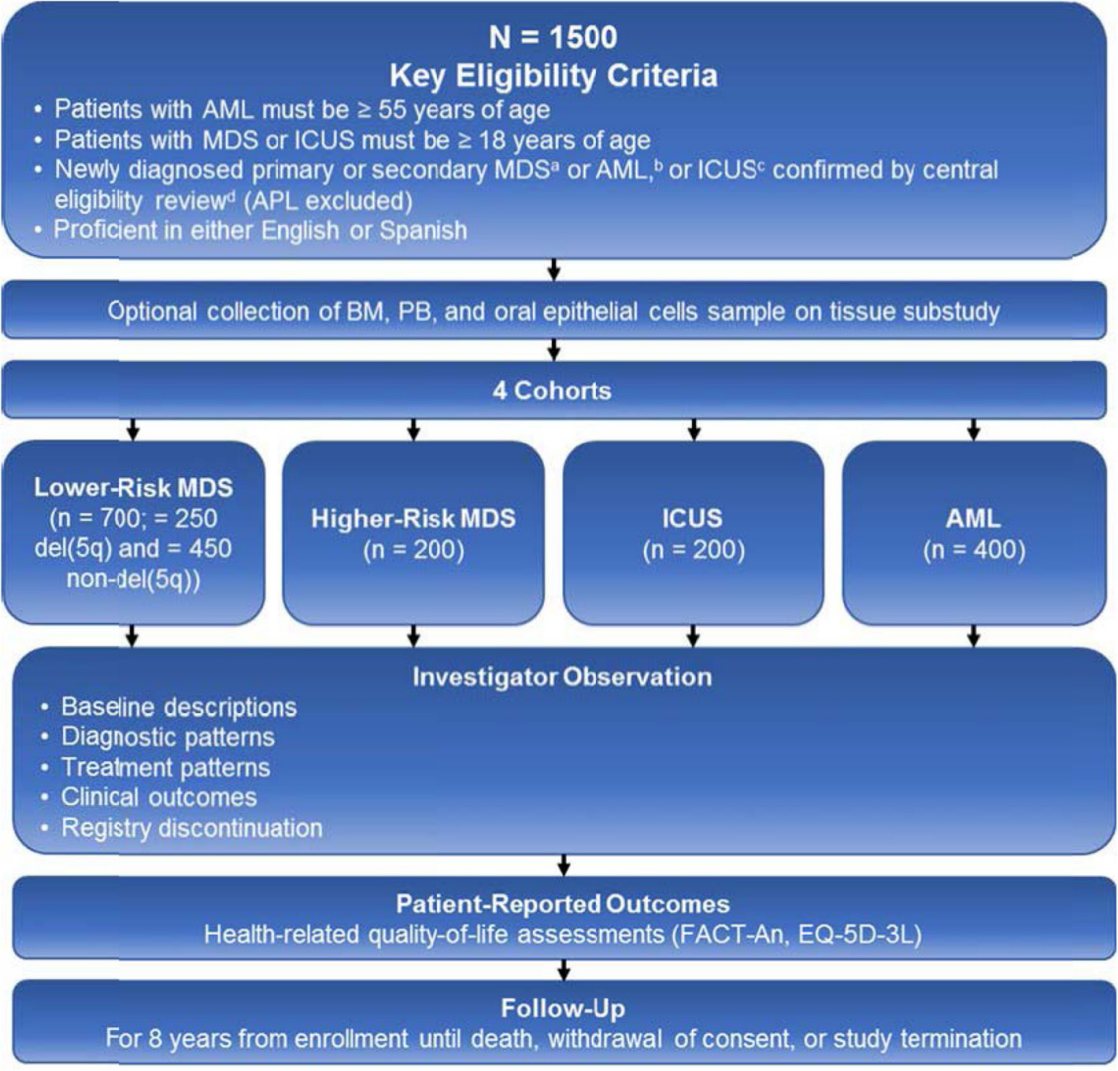
Connect MDS/AML Disease Registry study design. Overview of the study design of the disease registry from enrollment through follow-up. *AML* acute myeloid leukemia, *APL* acute promyelocytic leukemia, *BM* bone marrow, *EQ‐5D‐3L* EuroQOL. Group 5‐dimension 3‐level questionnaire, *FACT‐An* Functional Assessment of Cancer Therapy‐Anemia, *ICUS* idiopathic cytopenia of undetermined significance, *MDS* myelodysplastic syndromes, *PB* peripheral blood. ^a^MDS diagnosis refers to the date of initial BM aspirate/biopsies for patients. ^b^AML diagnosis refers to the date of BM aspirate/biopsies or the date of initial PB sample that led to the suspecte diagnosis. ^c^ICUS diagnosis refers to patients with ≥ 6 months’ cytopenia in ≥ 1 myeloid lineage who do not meet the criteria for diagnosis of MDS. ^d^Review of BM aspirate/biopsies reports and cytogenetic report, PB laboratory results, or other reports that led to diagnosis of MDS or AML. Tissue samples are not reviewed; patients whose diagnosis and/or risk cannot be confirmed are deemed screen failures

Approximately 1500 patients with MDS, ICUS, or AML will be enrolled into 4 main cohorts (Fig. 1). The sizes of the cohorts were chosen to ensure adequate representation to address the critical objectives of the registry. The first cohort consists of 700 patients with lower-risk MDS, classified as IPSS low and int-1 risk, and is divided into subcohorts of patients with del(5q) (n = 250) and without del(5q) (n = 450). One of the objectives of the del(5q) cohort is to describe treatment outcomes in these patients. Currently, the only approved treatment specific to the del(5q) subgroup is lenalidomide, and approximately 50 % of patients with del(5q) continue past 3 cycles of therapy. Therefore, the expectation is that 125 patients will be on lenalidomide for more than 3 cycles. For the 250 del(5q) patients with an expected response rate of 57 % with a 10-mg dose, based on the MDS-003 and MDS-004 studies, the 2-sided 95 % confidence interval for this estimate is 0.51 to 0.63. For the 450 non-del(5q) patients, based on the MDS-005 study, > 60 % of patients, or approximately 270 patients, are expected to complete 4 cycles of therapy. With an expected response rate of 27 %, the 2-sided 95 % confidence interval is 0.23 to 0.31 and 122 patients are expected to respond, providing enough patients to explore predictors of response. The second cohort consists of patients with higher-risk MDS (n = 200), classified as IPSS int-2 or high risk. Two hundred patients should be an adequate sample size to describe diagnostic patterns and treatment effectiveness in this cohort. The third cohort consists of patients with ICUS (n = 200). No sample size estimation was done for this group since it is viewed as exploratory. The fourth cohort consists of patients with AML aged ≥ 55 years (n = 400). The age criterion was selected for patients with AML because of the complexities of treating older patients due to age-related comorbidities and increased vulnerability to therapeutic toxicities [5, 21, 26]. A sample size of 400 patients should be sufficient to characterize the complexities of treating these older patients.

### Participants

Patients with newly diagnosed primary or secondary MDS or AML, according to the 2008 revised World Health Organization criteria [6], or ICUS as defined by Valent et al. [8] are eligible for inclusion. Patients do not have to receive treatment to participate. Disease diagnosis must (1) be confirmed by independent central eligibility review of clinical diagnostic reports of bone marrow aspirates and biopsies, cytogenetic analyses, molecular testing, and laboratory results and (2) occur ≤ 60 days prior to giving informed consent. Cohort assignment, including IPSS risk for patients with MDS, will be confirmed by central review. Reports of bone marrow aspirates or biopsies must be available for patients with MDS or ICUS but not those with AML if the laboratory results show ≥ 20 % blasts in the peripheral blood. Patients with MDS or ICUS must be ≥ 18 years of age, and patients with AML must be ≥ 55 years of age. Patients with suspected or proven acute promyelocytic leukemia are excluded because these patients benefit from treatment with distinct regimens that lead to favorable outcomes [21]. Patients with MDS or ICUS previously treated with disease-modifying agents, including prior cytotoxic agents for MDS (drugs for other cancers are allowed), azacitidine, decitabine, lenalidomide, or targeted therapies (eg, FLT3 inhibitors), are excluded. Patients with AML can have initiated treatment with active agents within 14 days prior to providing informed consent. Prior use of supportive care, such as transfusions, antibiotics, iron chelators, erythropoiesis-stimulating agents or other hematopoietic growth factors, and tumor lysis prophylaxis is allowed. Patients with AML secondary to MDS could have received prior therapy with active agents for treatment of MDS. All patients must also be willing and able to complete the enrollment and follow-up PRO instruments in English or Spanish.

### Data collection

Patient data will be entered into the electronic data capture (EDC) system at screening, enrollment (ie, baseline), and approximately quarterly intervals throughout the duration of a patient’s participation. All decisions regarding patient care (treatment, response assessment, etc.) will be determined by the study clinician, as the disease registry is non-interventional. The EDC will capture clinical outcomes, and patients will be followed for 8 years or until early study termination, patient withdrawal, or death. For patients with MDS treated with supportive care alone, the median survival ranges from 0.4 years in the high-risk IPSS group to 5.7 years in the low-risk IPSS group [28]. For patients with AML, the 5-year survival rate is 27 %, whereas for older patients (75–84 years), the 1-year survival is 15 % [12, 29]. Thus, the 8-year follow-up period is an adequate length of time to acquire robust data on both the short- and long-term outcomes of patients with MDS, ICUS, and AML. Follow-up will continue regardless of whether patients remain on or discontinue treatment. The total study duration is approximately 11 years, including a 3-year screening and enrollment period and an 8-year follow-up period.

Information to be captured by the disease registry includes baseline characteristics, comorbidities, frailty evaluations, diagnostic testing results, treatment, clinical outcomes, HEOR, and HRQOL, as described in Table 1. Patient-reported HRQOL data will be collected using 2 instruments: the Functional Assessment of Cancer Therapy-Anemia (FACT-An), which assesses physical, social, family, emotional, and functional well-being, and fatigue- and anemia-related concerns [30] and the EuroQol Group 5-dimension, 3-level questionnaire (EQ-5D-3 L), which assesses mobility, self-care, usual activities, pain and discomfort, anxiety, depression, and overall health status [31]. All clinical outcomes will be assessed by the treating physician as would occur in routine clinical practice (Table 2) and captured using electronic case report forms.

**Table 1:** Information and assessments captured by the Connect MDS/AML Disease Registry

Type of Assessment	Variables
Baseline descriptions	• Patient eligibility • Patient demographics and medical history • Prior malignancies • ECOG performance status • Clinical frailty scale • Adult comorbidity evaluation (ACE-27) • Current and concomitant medications
Diagnostic patterns	• Central eligibility review results • Hematology/peripheral blood laboratory results • Bone marrow biopsies/aspirate reports • FISH analysis, flow cytometry, molecular analysis reports • MDS and AML diagnostic testing and prognostic classification^a^
Treatment patterns	• Physician’s therapeutic objective • MDS and AML therapy, including supportive care • Changes in MDS and AML therapies • Transfusion information • Transplant eligibility and history • Select concomitant medications
Clinical outcomes	• Select chemistry laboratory results and other laboratory results • Response assessments • Survival information • Select AEs • Events of interest^b^
HEOR and HRQOL	• Hospitalization (number, length of stay, treatments used, etc.) • Patient-reported HRQOL instruments

*AE* adverse event, *AML* acute myeloid leukemia, *CNS* central nervous system, *ECOG* Eastern Cooperative Oncology Group, *FISH* fluorescence in situ hybridization, *HEOR* health economics and outcomes research, *HRQOL* health-related quality of life, *ICUS* idiopathic cytopenia of undetermined significance, *IPSS* International Prognostic Scoring System, *MDS* myelodysplastic syndromes

^a^For MDS, IPSS is used for prognostication. For AML, both cytogenetic and molecular data are used for risk assessment

^b^MDS: second primary malignancies; AML: extramedullary progression (including CNS) and second primary malignancies; ICUS: progression to MDS or AML

**Table 2:** Key clinical outcomes captured by the Connect MDS/AML Disease Registry

Type of Assessment	Variables
Long-term effectiveness	• Overall survival • Progression-free survival • Time to progression to AML • AML-free survival
Short-term effectiveness	• Complete remission marrow • Partial remission marrow • Complete remission peripheral blood • Progressive disease • Transfusion independence • Erythroid response • Platelet response • Neutrophil response • Progression/relapse after hematologic improvement • Cytogenetic response
Safety	• Type, frequency, and duration and outcomes of SAEs • Onset of SPM and other events of interest • AEs that lead to treatment discontinuation • Deaths/reasons for deaths

*AE* adverse event, *AML* acute myeloid leukemia, *SAE* serious adverse event, *SPM,* second primary malignancy

### Data quality

Certain aspects of the disease registry were designed to mitigate potential biases that could affect data quality. To control for selection bias, all consecutive patients at each site who are diagnosed with MDS, ICUS, or AML and who are potentially eligible for the disease registry will be presented with the option of enrolling, until accrual is met. To ensure high-quality data collection, each site will participate in training specific to registries via investigator meetings, teleconferences, or webinars, and a site initiation visit via teleconference or webinar with United BioSource Corporation (UBC). Ongoing site support and continuing education will be provided throughout the duration of the study. Information bias will be prevented through the proper handling of missing data by the EDC system. A major advantage of using an EDC system for data collection is that it allows real-time, remote data quality control through edit checks and data queries that are automated based on validation rules programmed in advance. This eliminates the need for on-site monitoring, as the programmed validation rules will obtain immediate feedback if data are missing or unclear. For example, data fields will be programmed in a way that prevents leaving an entry blank, and error messages will be generated in real time if values entered outside of the preset range are detected.

### Biomarker tissue substudy

MDS and AML are known to have a complex genetic architecture [2, 6, 10, 21]. A number of clonal abnormalities are known to have prognostic import in MDS and AML and are incorporated into assessments of disease risk [19, 21]. However, there are many more for which prognostic or predictive impact remain to be determined or conclusively validated. For example, more than 40 recurrent somatic mutations are described in MDS and are organized into a number of biological pathways involving pre-mRNA splicing, epigenetic patterning (including DNA methylation, which influences gene expression), chromatin conformation, and genome stability [2, 10]. In up to 40 % of patients with ICUS, MDS-associated genetic mutations have been observed, but no data exist regarding prognostic implications, and the extent to which these patients overlap with MDS is unclear, since 10 % of patients aged > 70 years with normal blood counts also have MDS-associated genetic mutations and are said to have CHIP [9–11].

Therefore, an optional, non-interventional, correlative substudy will be conducted to explore the relationship between karyotypes, molecular markers, and clinical outcomes. Participating patients will provide a bone marrow sample, collected as part of routine medical care at screening or later if done prior to active therapy initiation, as well as peripheral blood samples collected at screening (also prior to active therapy initiation) and at clinically relevant post-baseline time points. To aid in the distinction of somatic variants from germline polymorphisms, oral epithelial cells will be collected. A best effort will be made to collect all of these samples from study sites participating in the biomarker tissue substudy. All participating sites will follow a study-specific laboratory manual to collect and ship the samples to Genoptix, a Clinical Laboratory Improvement Amendments (CLIA)-certified laboratory. The substudy objectives are to:

1. Evaluate DNA mutations for further prognostic classification of MDS and AML subtypes, and evaluate their potential impact on treatment options;
2. Summarize clinical status of patients with and without mutations;
3. Analyze the correlation between mutations and allele burden in bone marrow and peripheral blood samples.

### Data analysis

The data generated from the disease registry will describe diagnostic patterns, treatment decisions and responses, therapeutic regimens, and associated clinical, HRQOL, and HEOR outcomes. Routine clinical practice patterns will be compared with existing management guidelines (ie, NCCN). The disease registry will be managed by UBC with oversight by the MDS and AML SSCs. Celgene, in conjunction with the SSCs, will establish a uniform procedure for analyzing, publishing, and disseminating findings from the disease registry. Data from all study centers will be combined, and all analyses will be performed within the disease cohorts. The final analyses will be performed by cohort once all patients within a cohort have completed the study. Descriptive summary statistics will be calculated for the majority of data collected, including demographics, diagnosis and IPSS risk classification, baseline characteristics, medical history, prior therapy, concomitant medications, treatment regimens and exposure, safety outcomes, and HEOR. Treatment-effectiveness outcomes, including response rates, disease progression, overall survival, other clinical outcomes, and HRQOL will also be summarized. Potential confounders will be considered, such as patient’s age group, type of enrolling institution (academic vs community), geographic location, and other demographic and baseline factors, particularly those that represent patient’s medical history and socioeconomic background. All statistical analyses will be conducted using SAS version 9.2 or higher. Statistical testing will be conducted at the α = 0.05 (2-sided) significance level, and 2-sided *P*-values and confidence intervals will be reported. Specialized methods, such as propensity score modeling, will be utilized to compensate for expected biases within these nonrandomized groups.

### Status of the registry

**Fig. 2:**
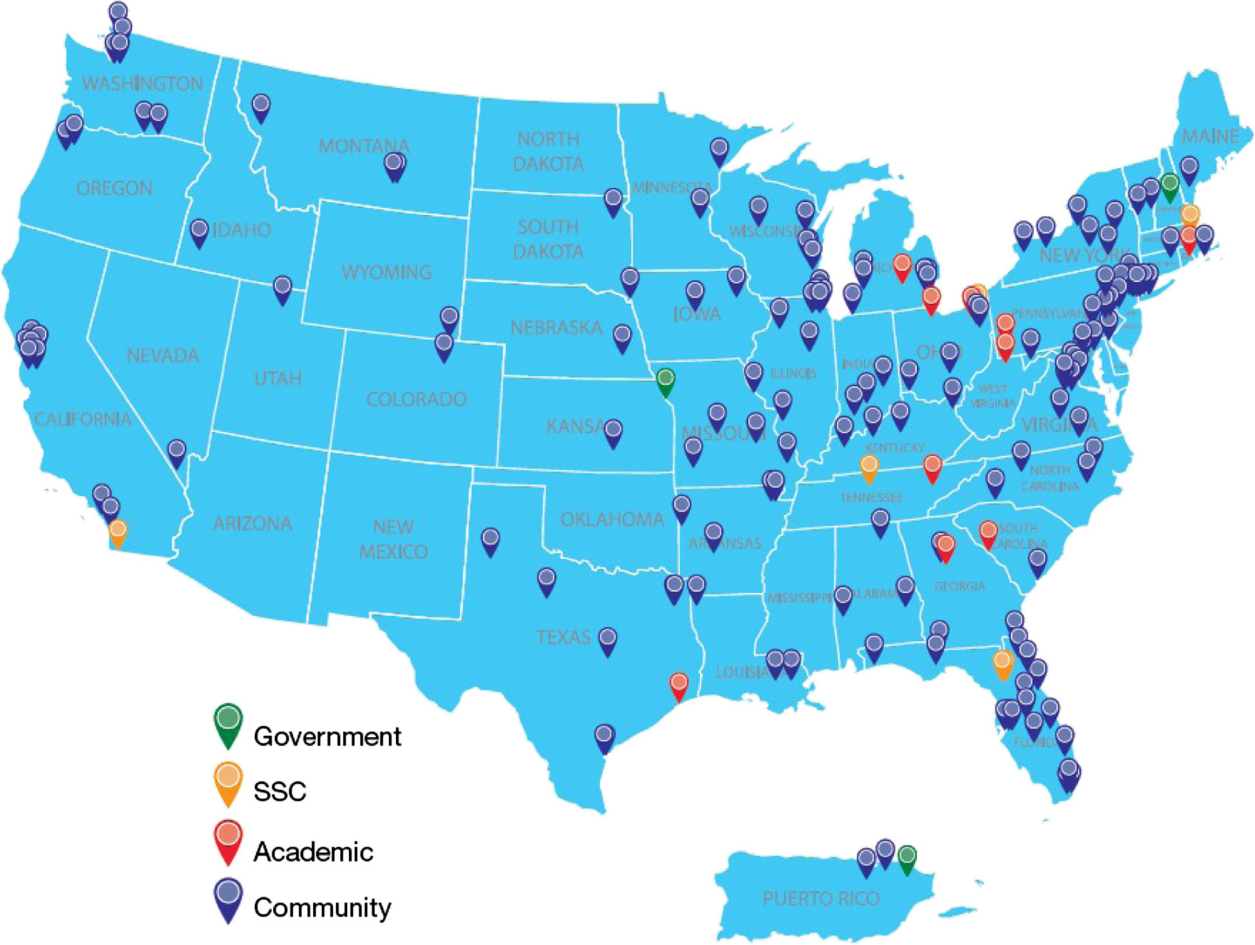
Locations of accepted study sites. As of November 30, 2015, there were 169 accepted study sites in the United States and Puerto Rico, including academic, community, and government sites

The first patient was enrolled in the disease registry on December 12, 2013. As of November 30, 2015, enrollment is 306 patients. There are currently 104 patients enrolled in the IPSS lower-risk MDS cohort (10 patients with del(5q) and 94 patients without del(5q)), 80 patients in the IPSS higher-risk MDS cohort, 0 patients in the ICUS cohort, and 121 patients in the AML cohort. There are currently 169 accepted study sites and 149 activated sites (Fig. 2).

## Discussion

For patients with MDS and AML, key variables affecting disease outcomes and survival, such as diagnostic trends, prognostic characterization, treatment patterns, and PROs, are inadequately documented outside of clinical trials. Even less is understood about patients with ICUS due to the recent definition of this population and its heterogeneity. As a result, decisions about treatment and management of patients with MDS, ICUS, and AML are complex, challenging, and complicated by elderly age, high frequency of comorbid conditions, and poor performance status or HRQOL [5, 11, 24]. To better understand the epidemiology, disease course, and long-term outcomes, this first prospective disease registry of patients with MDS, ICUS, or AML has been designed and initiated to capture longitudinal data for a cohort of patients within a single database.

The Connect MDS/AML Disease Registry represents an opportunity to synthesize information from several domains, including clinical parameters, diagnostic practices, prognostic classifications treatments patterns, treatment outcomes, and molecular data in a prospective fashion. The inclusion of data from such varied domains will contribute to a large, rich database for future analyses. Results may provide new insights into diagnostic patterns, treatment regimens, and treatment sequencing, as well as how these are associated with clinical outcomes in patients with MDS, ICUS, or AML in the United States who are treated outside the context of a clinical trial. The results will also facilitate evaluation of HRQOL and HEOR outcomes that may be associated with current treatment regimens in routine clinical practice in the United States. Correlative analyses using molecular data will increase the understanding of MDS, ICUS, and AML and may reveal novel prognostic and predictive biomarkers for these diseases and offer the opportunity to validate newly discovered biomarkers. As a recent update of the IPSS-R incorporates mutation data, it will be important to analyze the impact of these specific prognostic mutations on MDS cohorts included this registry. Given the ever-increasing information on molecular mutations in MDS and AML, this study will aid in describing mutations in the context of patient diagnosis, treatment, and outcomes.

## Additional file

Additional file 1: Ethics Committees/Review Boards Which Approved the Connect MDS/AML Study. A list of all Institutional Review Boards that have approved the Connect MDS/AML Disease Registry study. (DOCX 17 kb)

## Abbreviations

AML: Acute myeloid leukemia
CHIP: Clonal hematopoiesis of indeterminate potential
EDC: Electronic data capture
FACT-An: Functional Assessment of Cancer Therapy-Anemia
HEOR: Health economics and outcomes research
HMA: Hypomethylating agent
HRQOL: Health-related quality of life
ICUS: Idiopathic cytopenia of undetermined significance
Int-1: Intermediate 1 risk
Int-2: Intermediate 2 risk
IPSS: International Prognostic Scoring System
IPSS-R: Revised International Prognostic Scoring System
MDS: Myelodysplastic syndromes
NCCN: National Comprehensive Cancer Network
PRO: Patient-reported outcome
SSC: Scientific steering committee
UBC: United BioSource Corporation
