# Supplementary material for: Connect MDS/AML: design of the myelodysplastic syndromes and acute myeloid leukemia disease registry, a prospective observational cohort study
Source: BMC Cancer. 2016 Aug 19;16:652. doi: 10.1186/s12885-016-2710-6 (PMC4991094; doi:10.1186/s12885-016-2710-6)
Supplement: Additional file 1: — Ethics Committees/Review Boards Which Approved the Connect MDS/AML Study. A list of all Institutional Review Boards that have approved the Connect MDS/AML Disease Registry study. (DOCX 17 kb) [file 12885_2016_2710_MOESM1_ESM.docx]

**Additional File 1. Ethics Committees/Review Boards Which Approved the Connect MDS/AML Study**

| **Study Site Ethics Committee/Review Board** |
| --- |
| Alta Bates Summit Institutional Review Board |
| Baptist Health Lexington - IRB |
| Biomedical Research Alliance of New York Institutional Review Board |
| Broward Health Medical Center Institutional Review Board |
| Cape Cod Healthcare IRB |
| Cleveland Clinic Foundation IRB |
| Committee for the Protection of Human Subjects |
| Dana-Farber Cancer Institute, Office for Human Research Studies |
| DCH Health System |
| Dignity Health Institutional Review Board |
| Dignity Health Sacramento Regional IRB |
| Englewood Hospital & Medical Center IRB |
| Essentia Health Institutional Review Board |
| Glens Falls Hospital |
| Ingalls Memorial Hospital Institutional Review Board |
| IU Health Bloomington IRB |
| John Muir Health Institutional Review Committee |
| Kansas City Veterans Affairs Medical Center IRB |
| Lenoir Memorial Hospital |
| Liberty IRB |
| Maimonides Medical Center Institutional Review Board |
| Mary Greeley Medical Center IRB |
| Medstar Health Research Institute-Georgetown University Oncology IRB (Multiple Sites) |
| Memorial Healthcare System IRB |
| Mercy Hospital Fort Smith IRB |
| Michigan State University BIRB |
| Missouri Baptist Medical Center Institutional Review Board |
| New England Institutional Review Board |
| New York Medical College Committee for Protection of Human Subjects |
| Northshore University Health System IRB |
| Orlando Regional Medical Center IRB |
| Park Nicollet Institute Institutional Review Board (Multiple Sites) |
| PeaceHealth System Institutional Review Board |
| Phoebe Putney Memorial Hospital IRB |
| ProHealth Care Internal Review Board |
| Providence Health & Services Institutional Review Board |
| Quorum Review IRB (Multiple Sites) |
| Rochester General Health System Clinical Investigation Committee |
| Roger Williams Medical Center Institutional Review Board |
| Rush University Medical Center IRB |
| Sacred Heart Health System Institutional Review Board |
| Saint Alphonsus Regional Medical Center Institutional Review Board |
| Salem Health Institutional Review Board |
| Scott & White Institutional Review Board |
| Siouxland IRB |
| Somerset Medical Center Institutional Review Board |
| St. Francis Hospital |
| St. Vincent Hospital IRB |
| St. Vincent Institutional Review Board |
| Summa Health System IRB |
| The Institutional Review Board at Tallahassee Memorial |
| The Mary Imogene Bassett Hospital Institutional Review Board |
| UCSD Human Research Protections Program (HRPP) |
| University & Medical Center Institutional Review Board |
| University Hospitals Case Medical Center IRB |
| University of Minnesota Human Research Protection Program |
| UTMB Institutional Review Board |
| Vanderbilt Human Research Protection Program |
| West Michigan Cancer Center IRB |
| West Virginia University Office of Research Integrity and Compliance |
| Western Institutional Review Board (Multiple Sites) |
| Wheaton Franciscan Healthcare IRB (Multiple Sites) |
